# Supplementary material for: Disentangling Crocus Series Verni and Its Polyploids
Source: Biology (Basel). 2023 Feb 14;12(2):303. doi: 10.3390/biology12020303 (PMC9953621; doi:10.3390/biology12020303)
Supplement: Supplementary file 1 [file biology-12-00303-s001.zip › R2_Suppl-Fig.pdf]

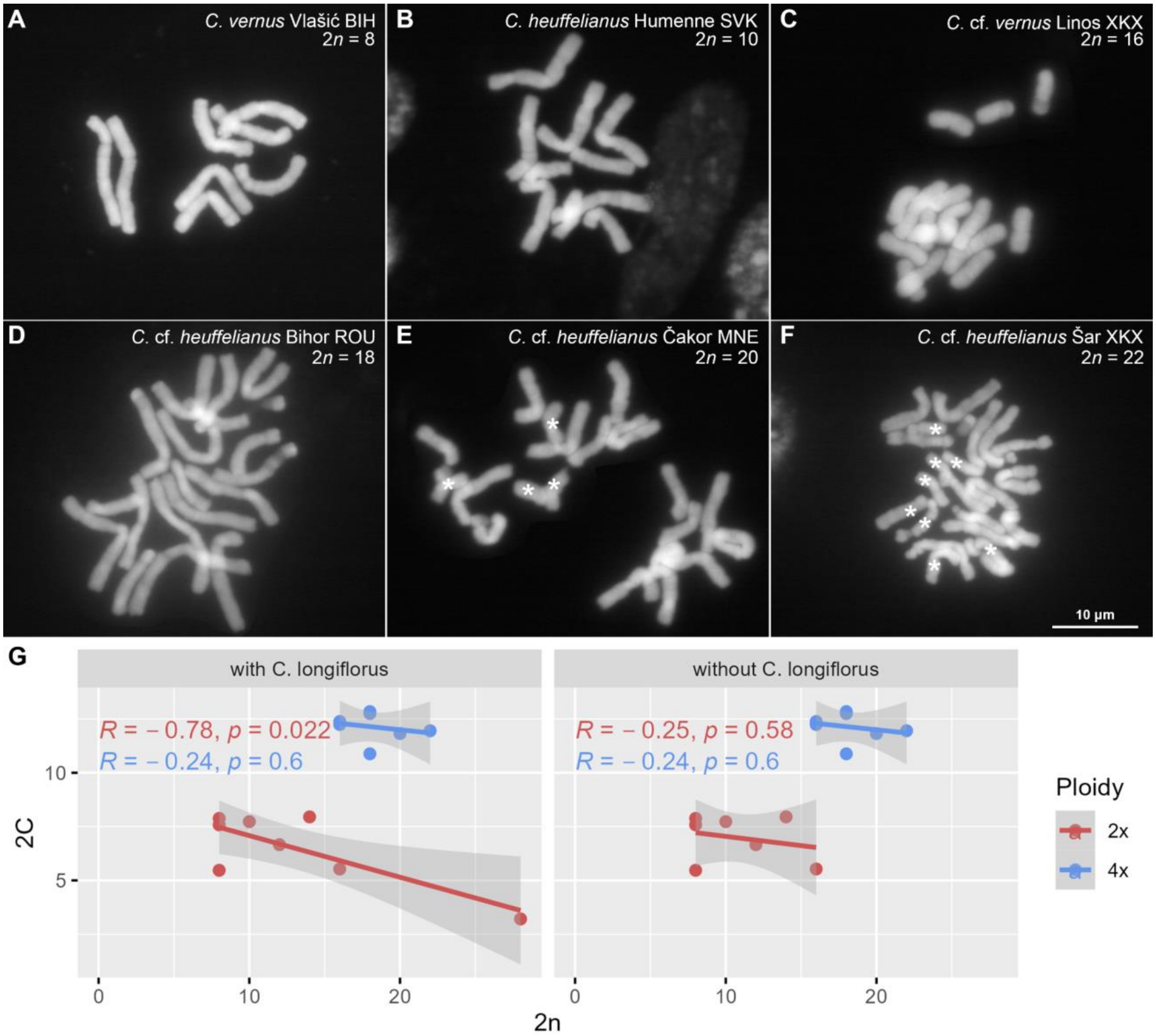

Figure S1. Chromosome counts for diploid *C. vernus* and *C. heuffelianus* (A and B), tetraploid *C.* cf. *vernus* (C), and tetraploid *C.* cf. *heuffelianus* (D-F). Asterisks show relatively shorter chromosomes. G) The chromosome number and genome size in diploid and tetraploid *C.* ser. *Verni* taxa show a general negative relationship, which was only significant when *C. longiflorus* was included.

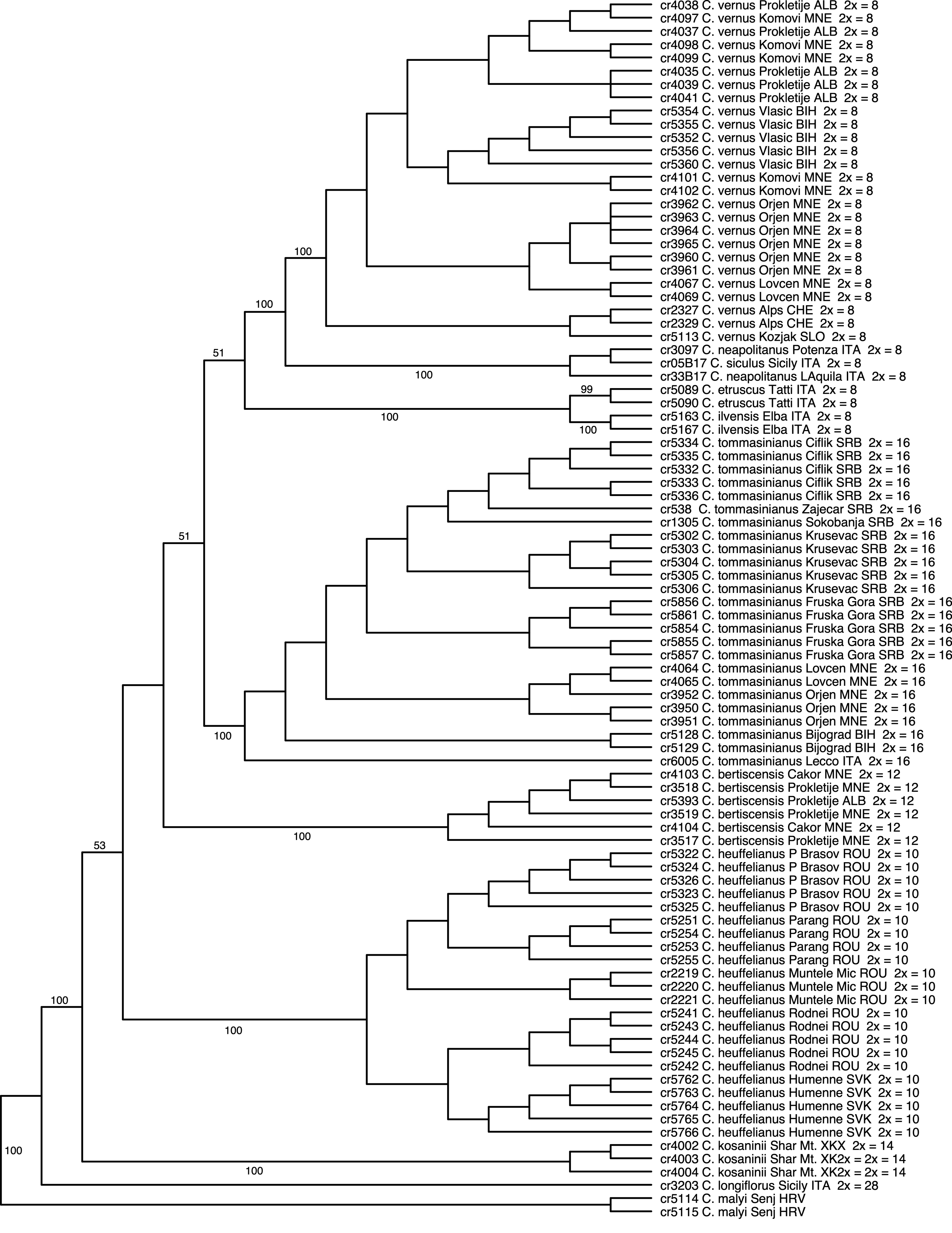

Figure S2. Strict consensus MP tree based on 2009 GBS loci including only diploid accessions of *Crocus* ser. *Verni* taxa.

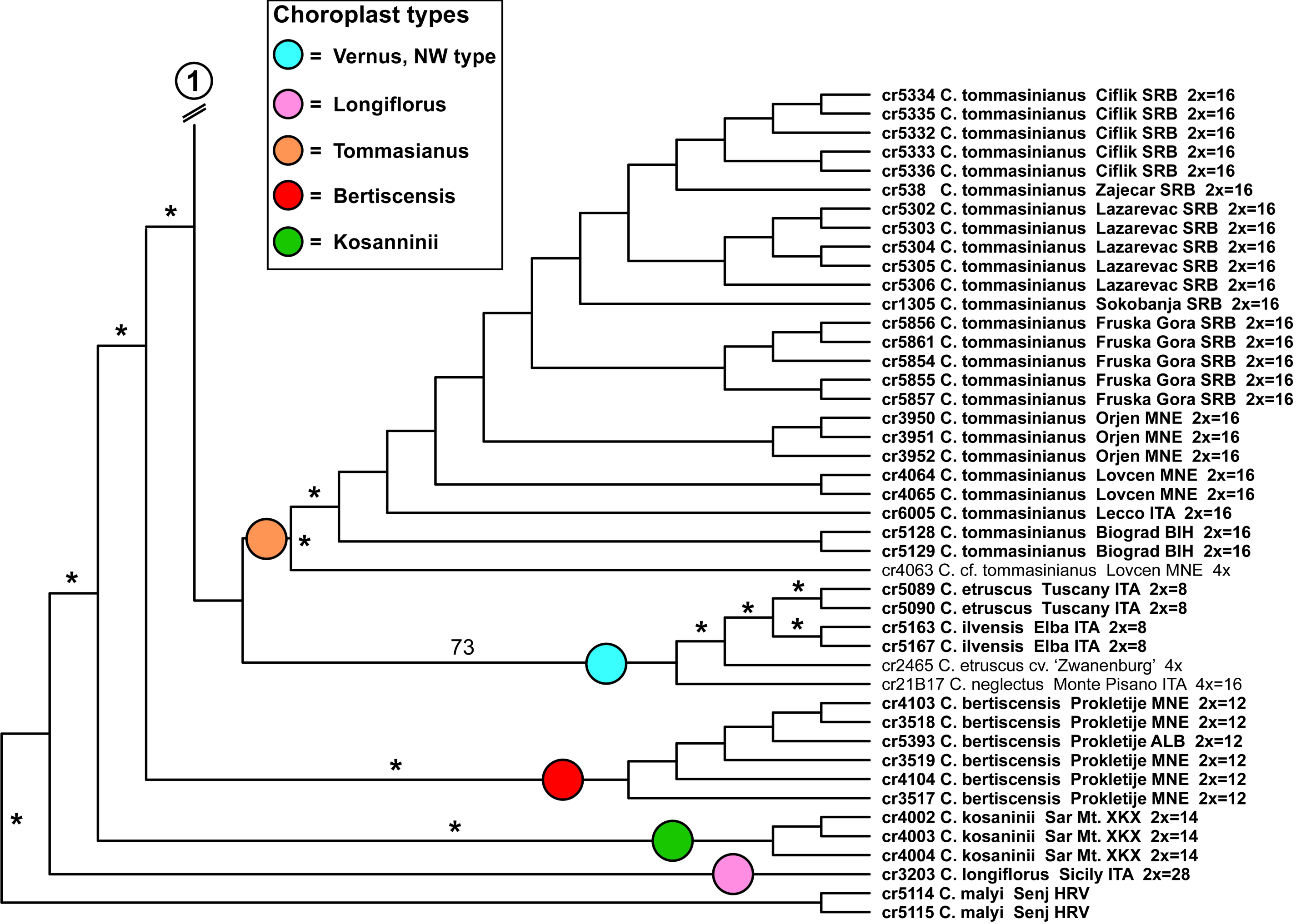

Figure S3. Strict consensus of 4500 most parsimonius phylogenetic trees derived from an analysis of the GBS dataset including di- and tetraploid cytotypes of *Crocus* ser. *Verni* taxa. Numbers along the backbone branches provide bootstrap values (≥50%) with asterisks indicating support values >80%. Colors in the circles refer to the chloroplast types present in the respective clades. Bold face indicate diploid individuals. *Crocus malyi* was defined as outgroup in the analysis.

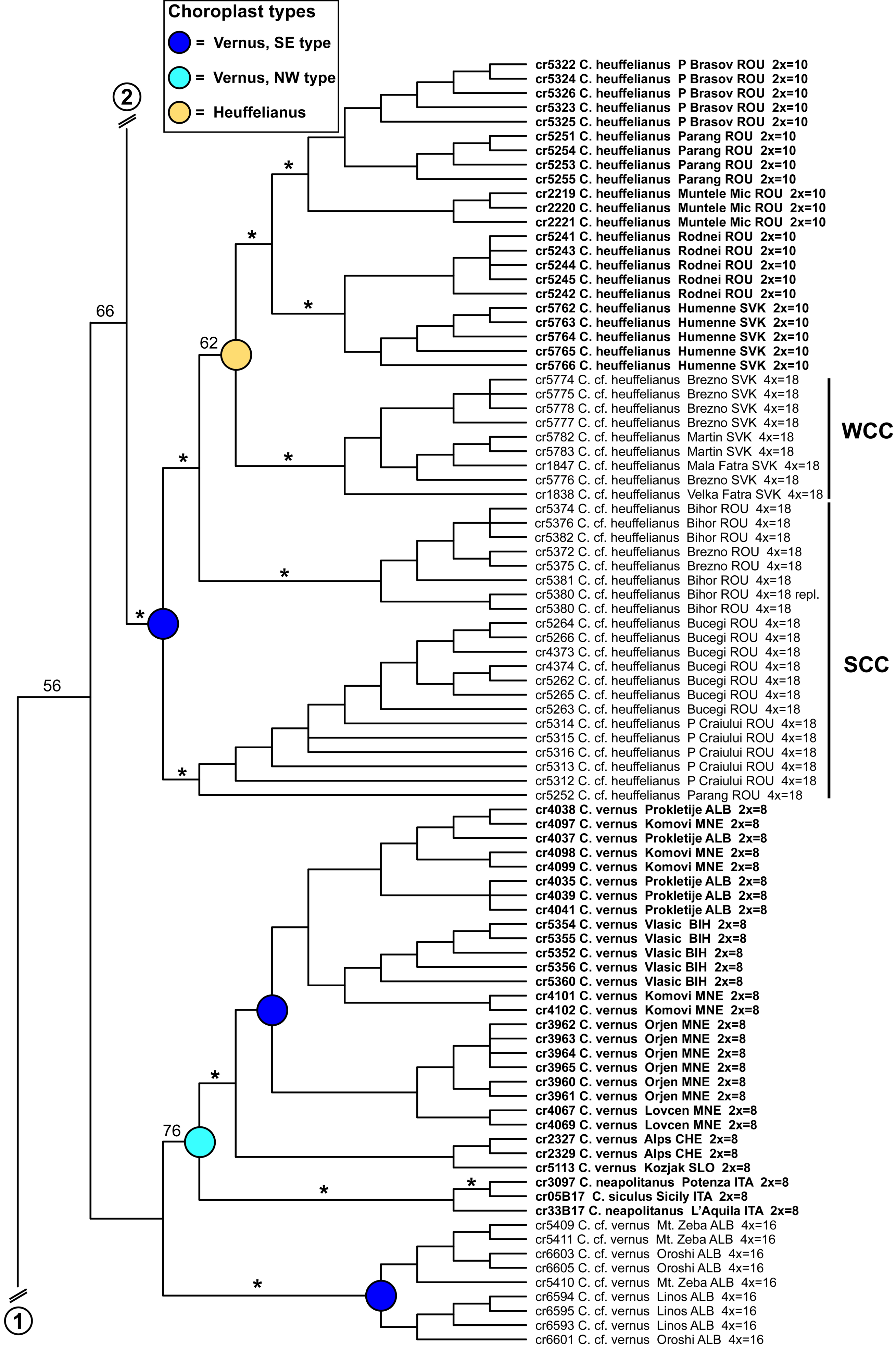

Figure S3. Continued.

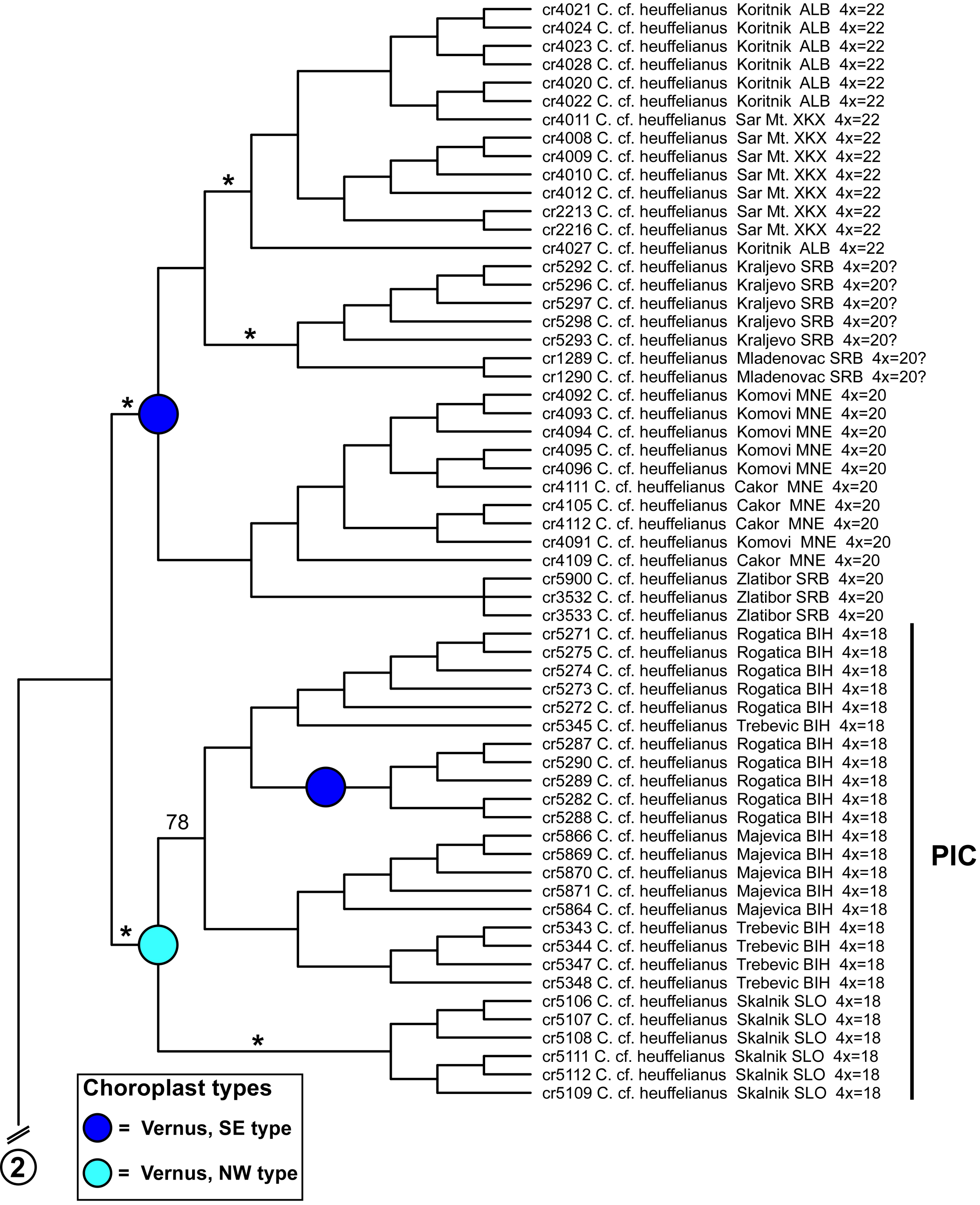

Figure S3. Continued.

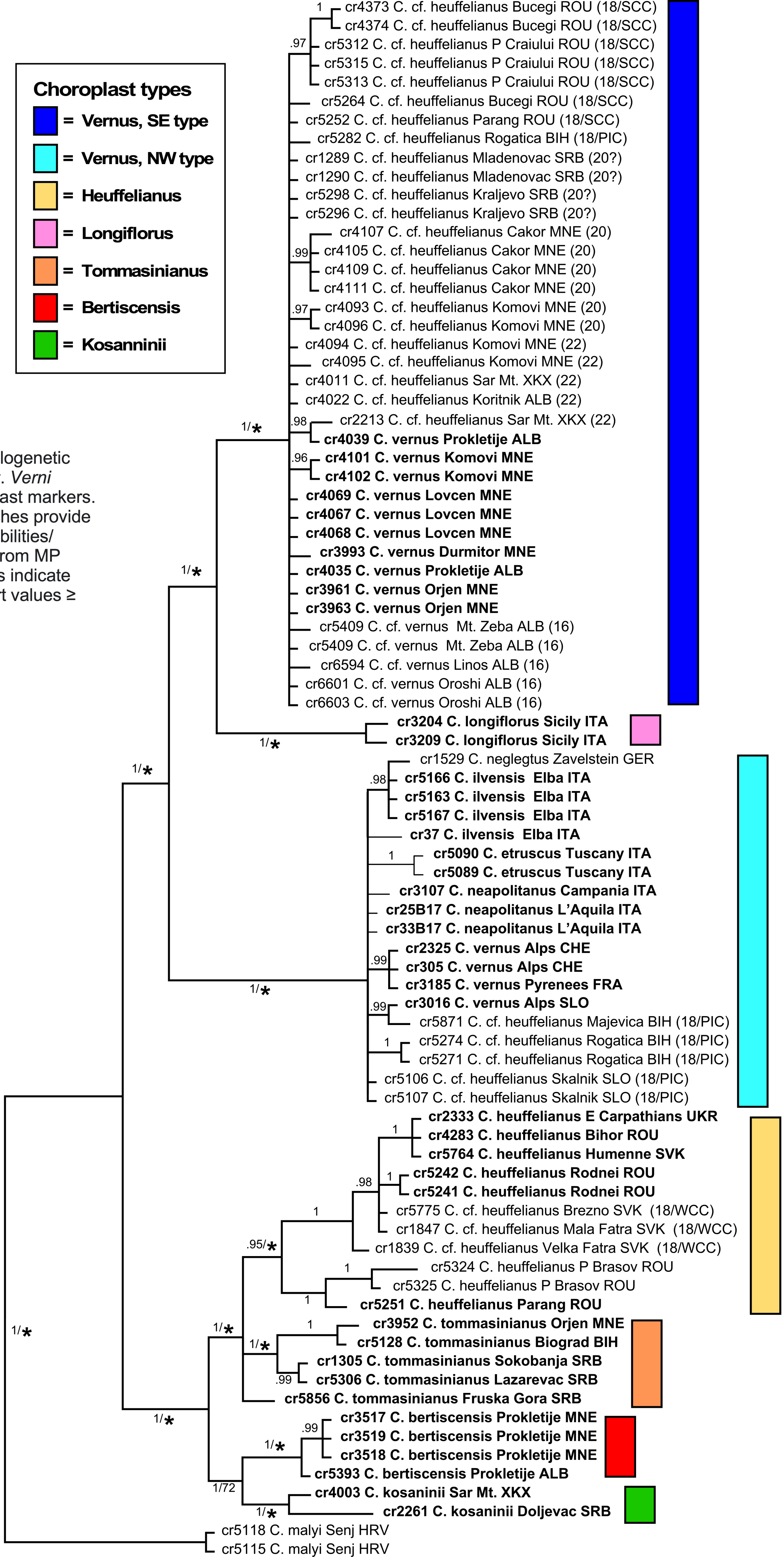

Figure S4. BI phylogenetic tree of *Crocus* ser. *Verni* based on chloroplast markers. Numbers at branches provide BI posterior probabilities/ bootstrap values from MP analysis. Asterisks indicate bootstrap ssupport values ≥ 80%.

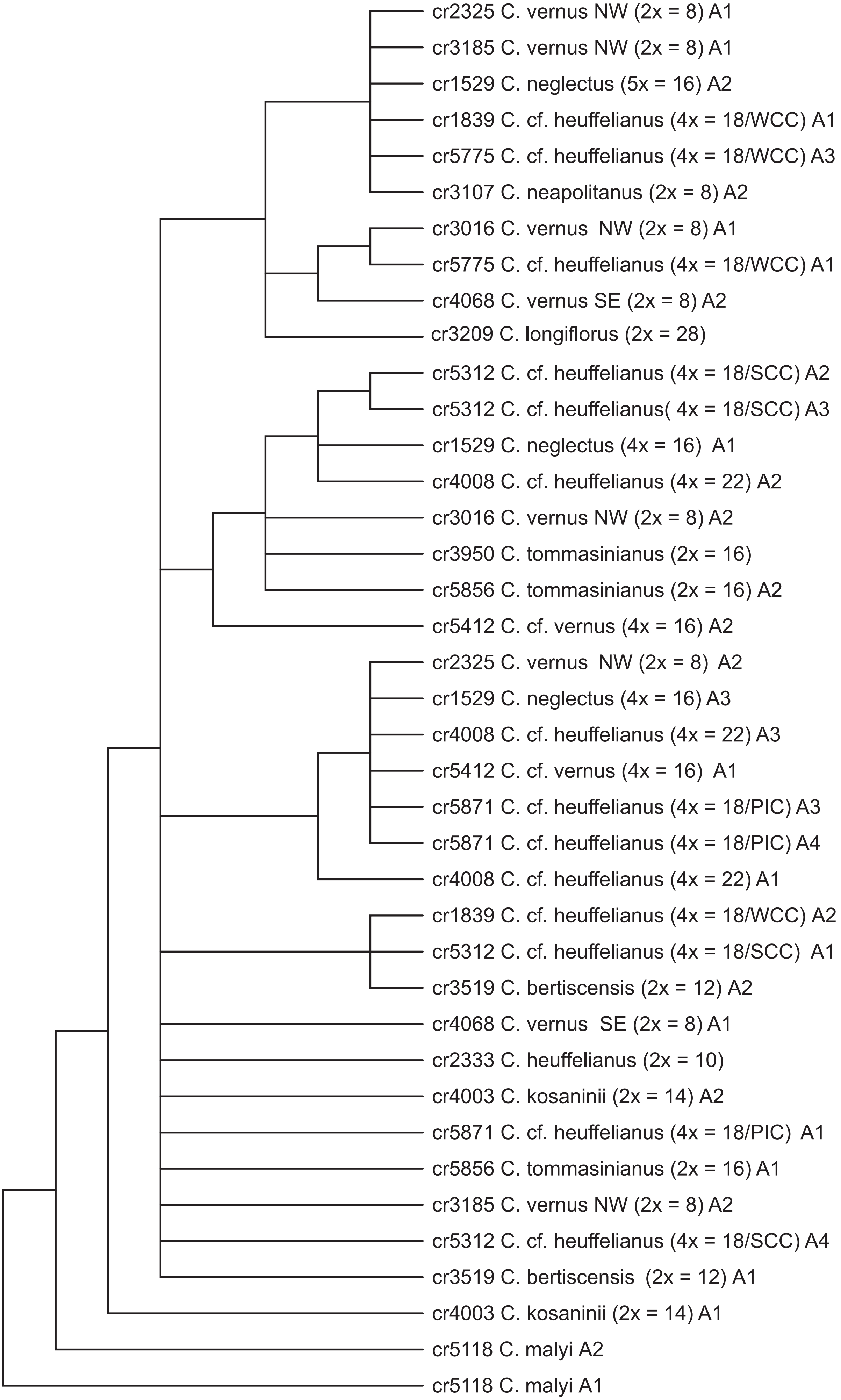

Figure S5. Strict consensus MP tree of *topo6*. Allelic differences (A1–A4) in these markers were used to track the bi-parental contributions of diploids to allotetraploids.

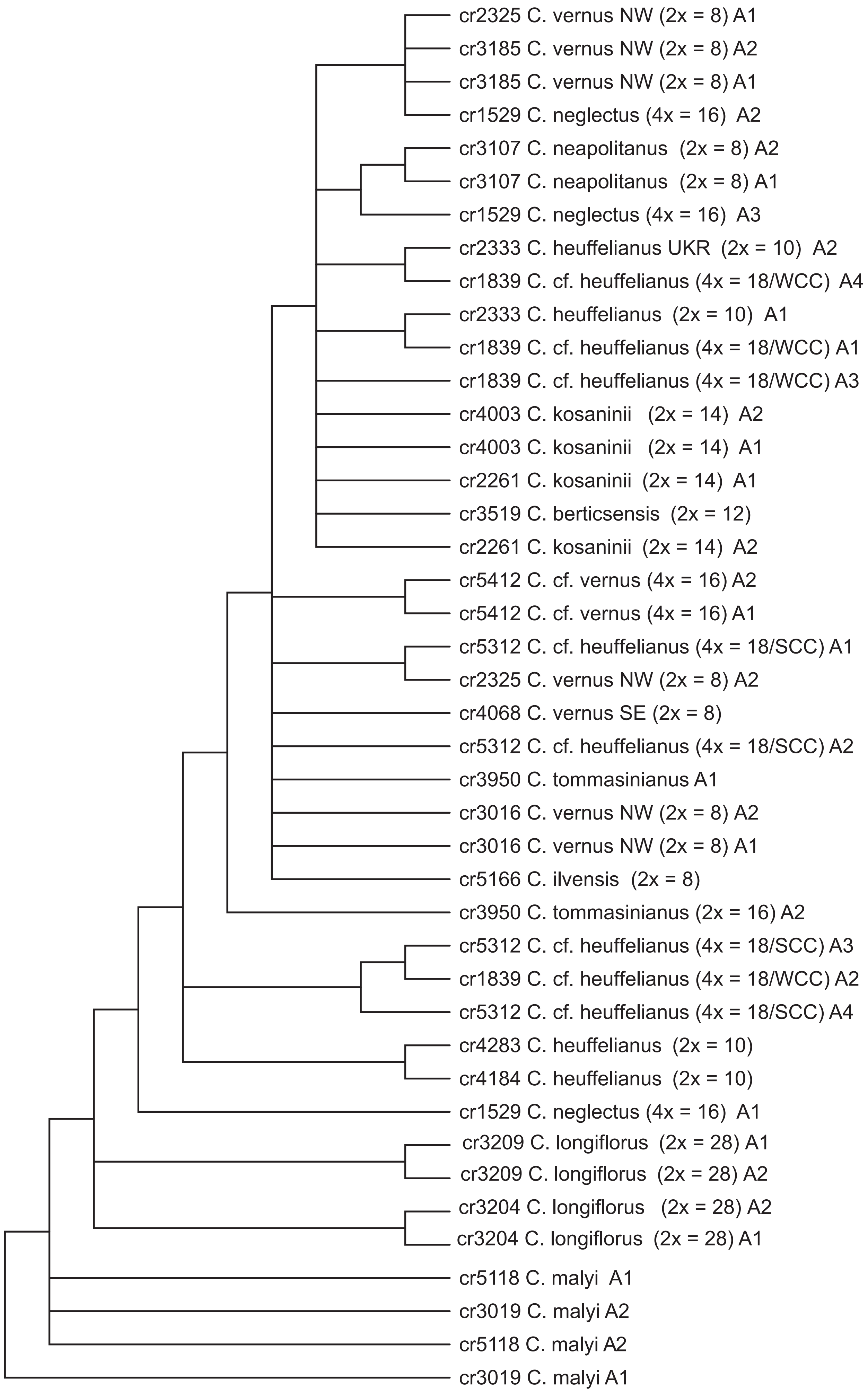

Figure S6. Strict consensus MP tree *rcf*2. Allelic differences (A1–A4) in these markers were used to track the bi-parental contributions of diploids to allotetraploids.

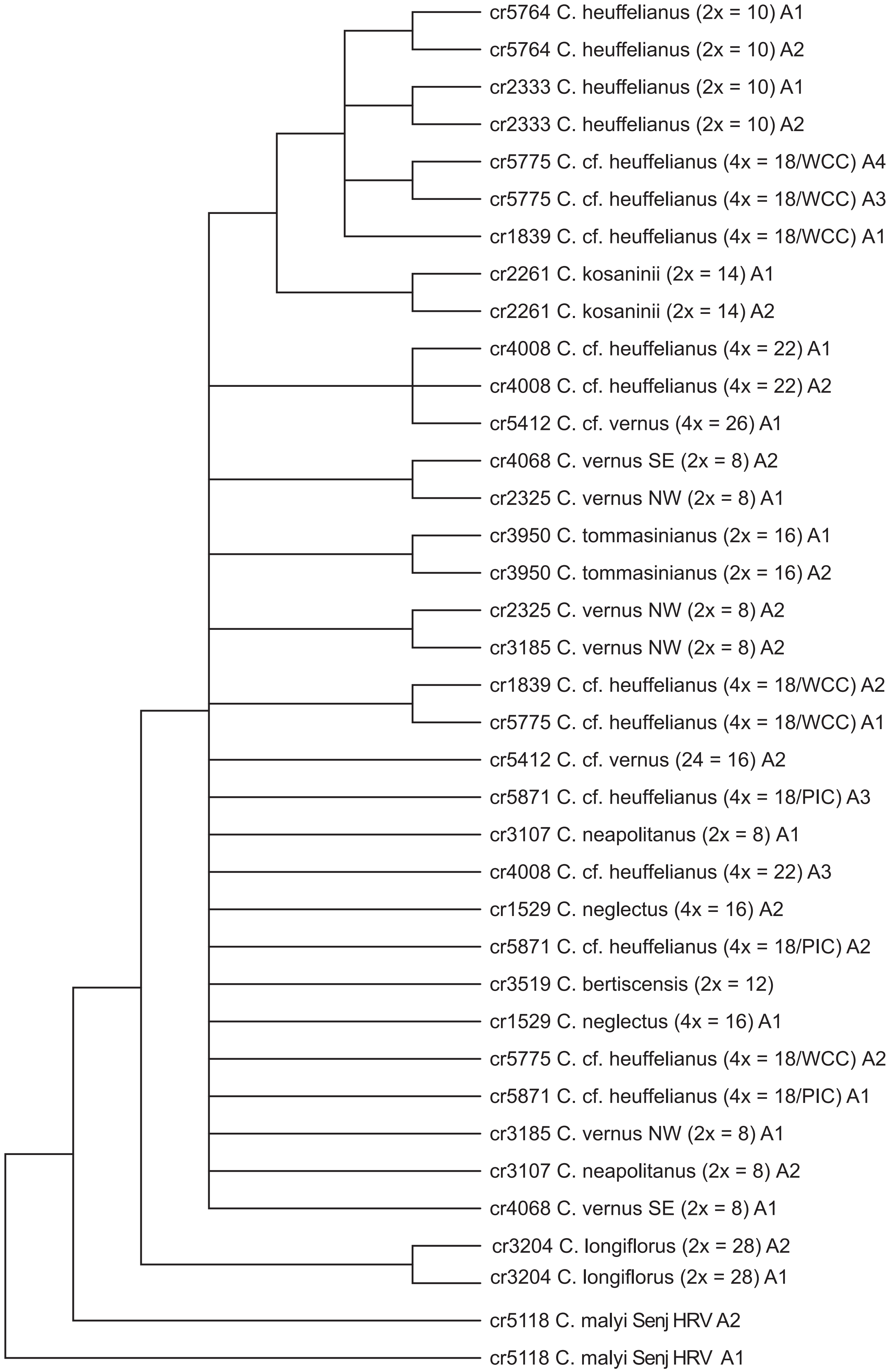

Figure S7. Strict consensus MP tree *orcp*. Allelic differences (A1–A4) in these markers were used to track the bi-parental contributions of diploids to allotetraploids.

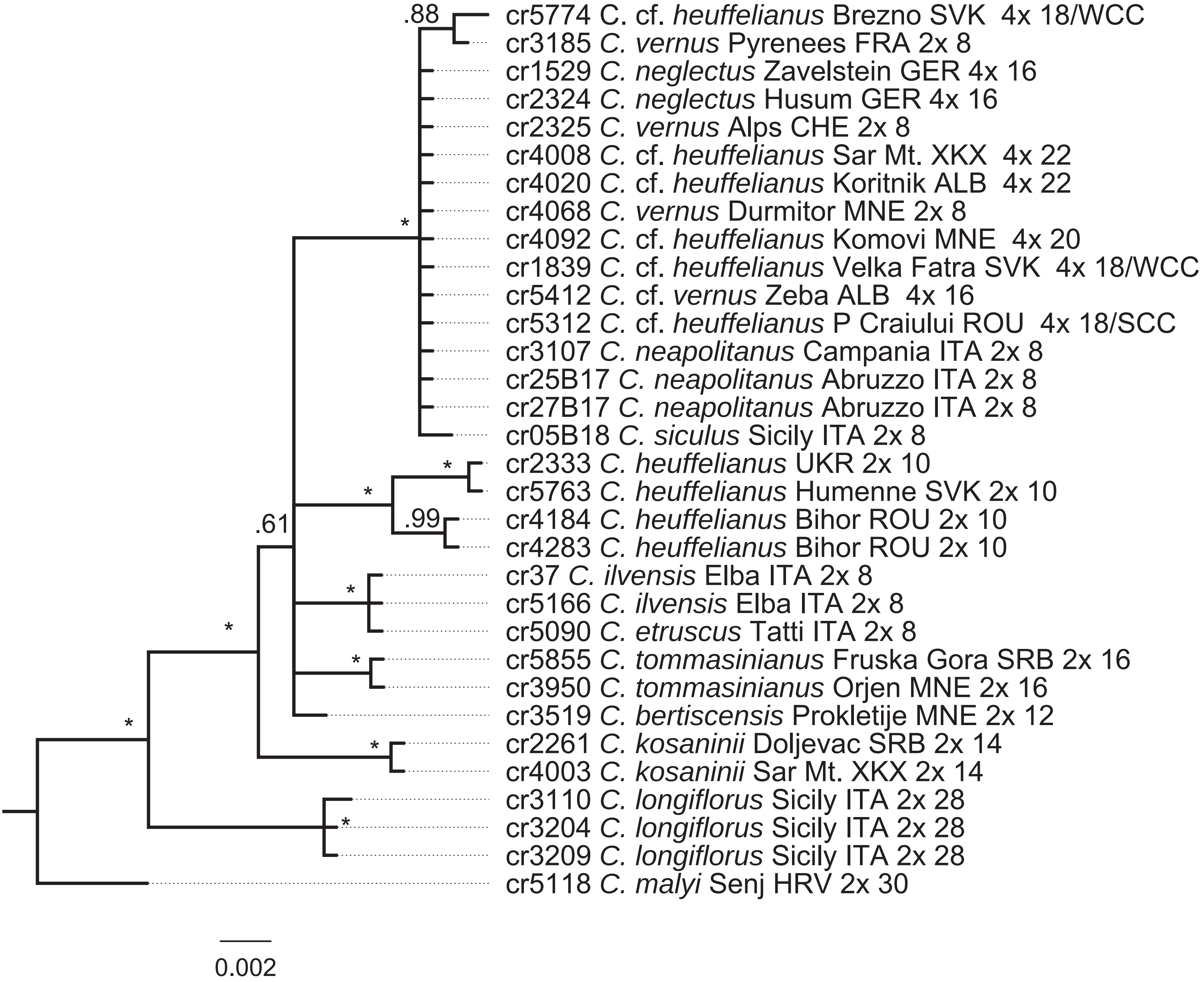

Figure S8. Phylogenetic trees obtained through Bayesian phylogenetic inference based on rDNA ITS sequences. Numbers along branches indicate BI posterior probabilities (pp), pp supports of 1.0 are indicated by asterisks.

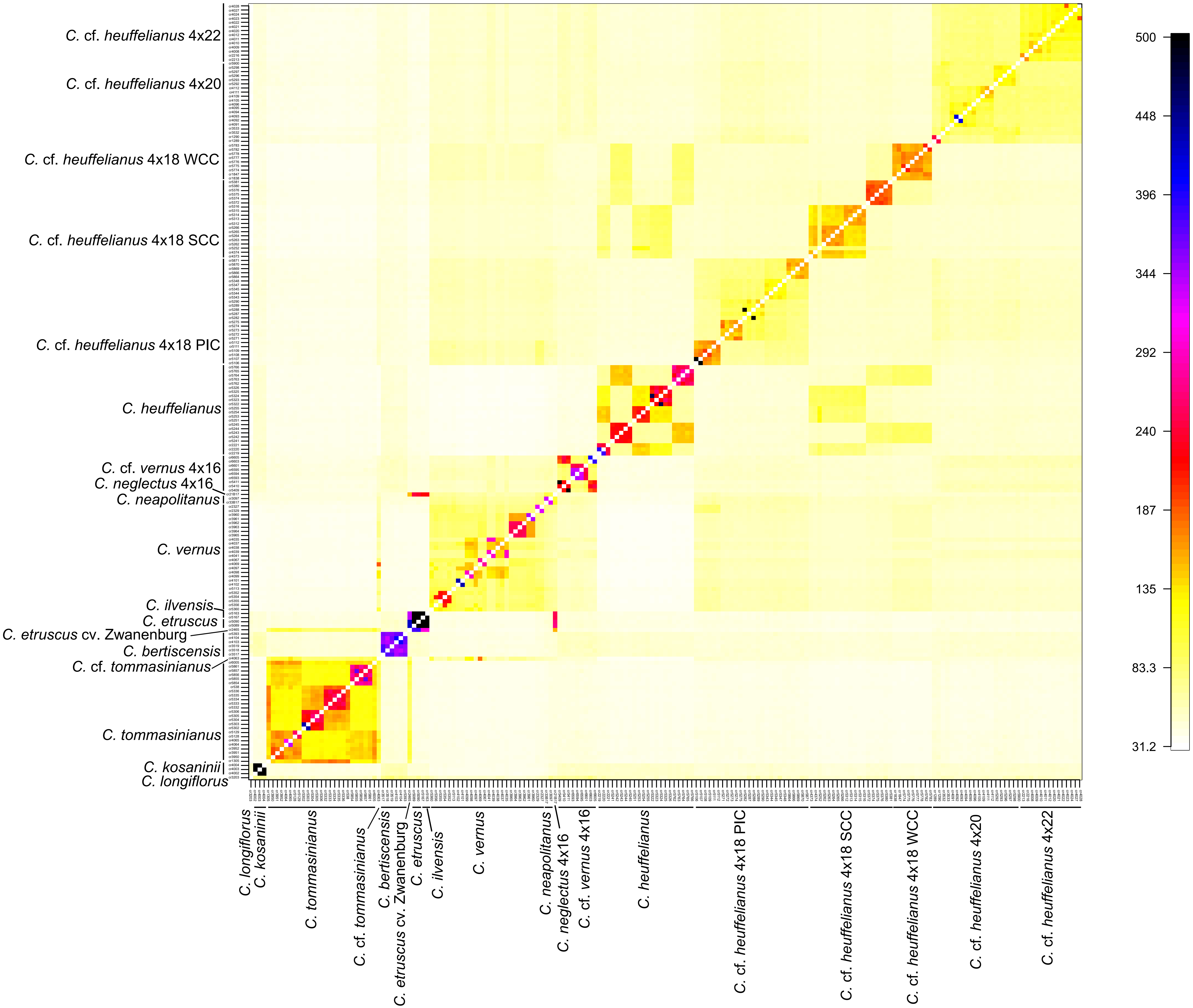

Figure S9. FineRADstructure co-ancestry matrices of the study species (without outgroup species *C. malyi*; *C. siculus* was excluded due too low coverage). Black indicates maximum levels of co-ancestry between two individuals, white the minimum (scale on the right). Numbers below the plots indicate the sample ID.

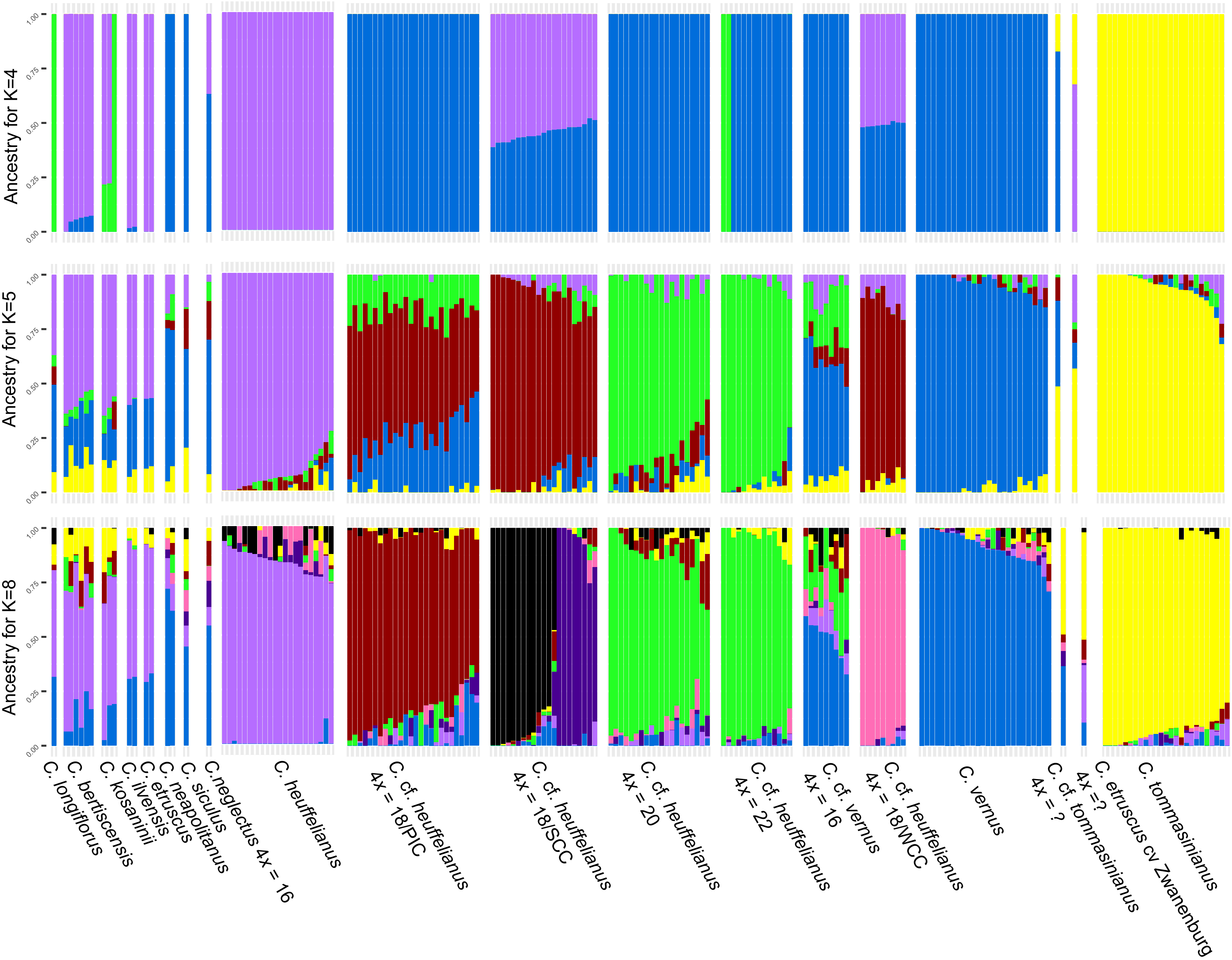

Figure S10. Population structure analysis based on 2207 GBS loci using fastSTRUCTURE at K = 4 (30661 SNPs) and LEA K = 5 and 8 (2172 unlinked SNPs). Each vertical line represents one individual, while each color shows the genetic composition that is assigned into a distinct genetic cluster.

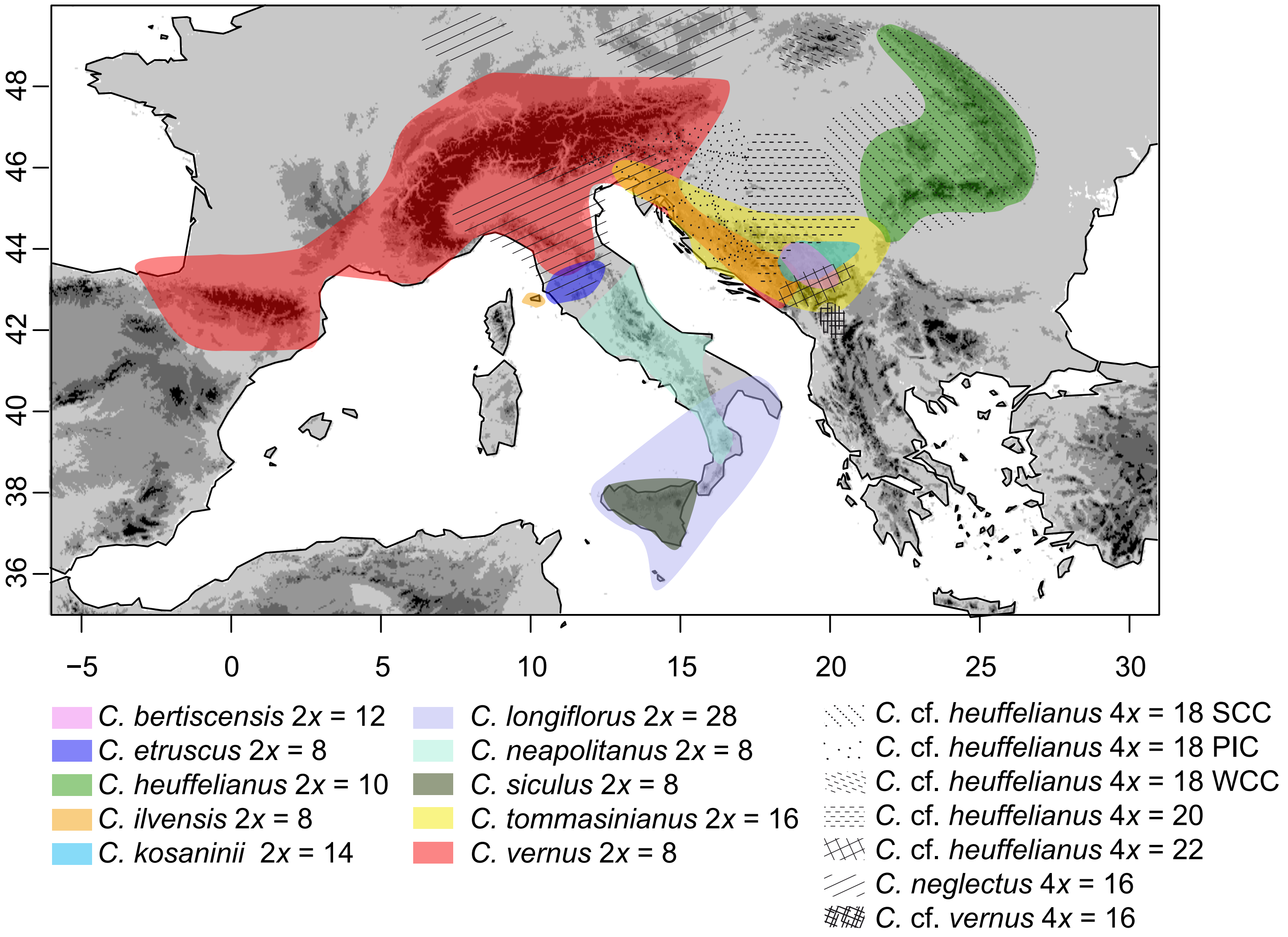

Figure S11. Map with the approximate distributions of species in *Crocus* ser. *Verni*. Distribution areas of different species are indicated by different colors or shapes (see legend).
